# Up-regulation of miR-155 potentiates CD34+ CML stem/progenitor cells to escape from the growth-inhibitory effects of TGF-ß1 and BMP signaling

**DOI:** 10.17179/excli2021-3404

**Published:** 2021-04-15

**Authors:** Touba Mahdloo, Pantea Sahami, Reihaneh Ramezani, Mojtaba Jafarinia, Hamedreza Goudarzi, Sadegh Babashah

**Affiliations:** 1 Department of Genetics, Faculty of Basic Sciences, Islamic Azad University, Marvdasht, Iran; 2 Department of Biomedical Sciences, Women Research Center, University of Alzahra, Tehran, Iran; 3 Department of Molecular Genetics, Faculty of Biological Sciences, Tarbiat Modares University, Tehran, Iran

**Keywords:** chronic myeloid leukemia, bone morphogenetic protein, TGF-beta signaling, miR-155

## Abstract

microRNAs (miRNAs or miRs) play key roles in different stages of chronic myeloid leukemia (CML) pathogenesis. The present study aimed to demonstrate whether miR-155 enables CD34^+^ CML cells to escape from the growth-inhibitory effects of TGF-β1 and bone morphogenetic protein (BMP) signaling. Among differentially expressed miRNAs in CD34^+^ CML cells, miR-155 was highly up-regulated. QRT-PCR revealed an inverse correlation between miR-155 and two key members of the TGF-β pathway-TGF-βR2 and SMAD5. Results showed that SMAD5 is not only up-regulated through BMPs treatment, but recombinant TGF-β1 can also induce SMAD5 in CML cells. We also demonstrated that TGF-β1-mediated phosphorylation of SMAD1/5 was abolished by pre-treatment with the blocking TGF-βR2 antibody, suggesting a possible involvement of TGF-βR2. Additionally, overexpression of miR-155 significantly promoted the proliferation rate of CD34^+^ CML cells. Results showed that siRNA-mediated knockdown of SMAD5 had a promoting effect on CD34^+^ CML cell proliferation, suggesting that SMAD5 knock-down recapitulates the proliferative effects of miR-155. Importantly, TGF-β1 and BMP2/4 treatment had inhibitory effects on cell proliferation; however, miR-155 overexpression enabled CD34^+^ CML cells to evade the anti-proliferative effects of TGF-β1 and BMPs. Consistently, down-regulation of miR-155 augmented the promoting effects of TGF-β1 and BMP signaling on inducing apoptosis in CD34^+^ CML stem cells. Our findings demonstrated that targeting of SMAD5 and TGF-βR2 links miR-155 to TGF-β signaling in CML. Overexpression of miR-155 enables CD34^+^ CML cells to evade growth-inhibitory effects of the TGF-β1 and BMP signaling, providing new perspectives for miR-155 as a therapeutic target for CML.

## Introduction

Chronic myeloid leukemia (CML) is a malignant hematological disease with an incidence of 1-2 cases per 100,000 individuals (Siegel et al., 2015[47]). CML is divided into three phases including Chronic Phase (CP), Accelerated Phase (AP), and Blastic Phase (BP) (Pasic and Lipton, 2017[38]). CML is characterized by a well-known chromosomal translocation, the Philadelphia chromosome or t(9; 22) (q34; q11), during which, a large part of the Abelson (*ABL1*) oncogene on chromosome 9 is translocated to the breakpoint cluster region (*BCR*) of the long arm of chromosome 22, resulting in a *BCR-ABL1* fusion gene. The presence of constitutively active tyrosine kinase BCR-ABL1 promotes multiple oncogenic signaling pathways leading to the CML clinical condition (Babashah et al., 2013[4]). Development of therapeutics designed to inhibit BCR-ABL such as tyrosine kinase inhibitors (TKIs) Imatinib and Dasatinib has improved the therapy of CP-CML; however, the emergence of leukemic stem cell (LSC) clones eventually relapse and succumb to the disease in CML patients in AP or BP (Chorzalska et al., 2018[12]). 

Different studies indicated that *BCR-ABL1* oncogene activates multiple signaling pathways promoting the differentiation and survival of LSCs mainly through Wnt/β-catenin, PI3K/AKT/mTOR, Hedgehog, and Notch signaling pathways (Aljedai et al., 2015[3]; Houshmand et al., 2019[20]; Jamieson et al., 2004[21]; Reya et al., 2003[44]). More specifically, TGFβ-Smad is another important signaling pathway, regulating the self-renewal properties of LSCs (Naka and Hirao, 2017[32]). TGFβ-Smad axis contains different bone morphogenetic proteins (BMPs) which they involve in CML pathogenicity. Unsurprisingly, aberrant regulation of these factors has been seen in CML (Zhang et al., 2016[57]). BMPs are a group of growth factors containing 15 proteins with six members (BMP2-7) belonging to the TGFβ superfamily (Firestein et al., 2016[15]). BMPs are implicated in several cellular processes including cellular differentiation, embryogenesis, organogenesis, and apoptosis (Nishimura et al., 2003[35]). Accordingly, dysregulation of these factors can essentially play roles in the etiology of various cancers including leukemia (Toofan et al., 2014[51]). Previous studies have revealed that the SMAD family is mainly responsible for mediating the biological effects of BMPs (Nishimura et al., 2003[35]).

So far, different conducted investigations have demonstrated that microRNAs (miRNAs or miRs) might have both oncogenic and tumor-suppressive functions in CML (Buhagiar et al., 2020[9]). This group of small single-strand non-coding RNAs plays a critical role in different aspects of tumor development to touch upon proliferation, angiogenesis, and apoptosis (Babashah and Soleimani, 2011[6]; Masoumi-Dehghi et al., 2020[28]). miR-155 is a well-known oncogenic miRNA which has been dysregulated in different cancer (Bitaraf et al., 2020[7]; Kapral et al., 2019[22]; Narayan et al., 2018[33]; Zhou et al., 2019[59]). This miRNA has been proven to target several mRNAs that contribute to leukemogenesis (Neilsen et al., 2013[34]). 

In the present study, we profiled the miRNA expression in CD34^+^ cells isolated from CML patients with the blastic phase phenotype as well as healthy individuals. We demonstrated that miR-155 is overexpressed in the patients' CD34^+^ CML cells than the normal cells. We also showed that SMAD5 is a potential direct target for miR-155 and is down-regulated in CD34^+^ CML cells. Furthermore, we indicated that the up-regulation of oncogenic miR-155 potentiates CD34^+^ CML stem cells to escape from the apoptotic and the growth-inhibitory effects of TGF-β1 and BMP signaling. 

## Materials and Methods

### Samples and cell line

Primary CD34^+^ cells were obtained by the bone marrow (BM) aspiration from five CML patients with blastic phase and five individuals without any hematological abnormalities. All participants provided written informed consent, and the study was approved by the ethics committee at each participating center. All of the CML patients had the Philadelphia chromosome, as confirmed by fluorescence in situ hybridization (FISH) analyses in addition to quantitative PCR. Mononuclear cells and CD34^+^ cells were isolated using Ficoll separation and magnetic-activated cell sorting (MACS) immunomagnetic separation system (Miltenyi Biotech, Germany), respectively. To assess the purity of CD34^+^ enriched cells, we used flow cytometric analysis whereby around 95 % of the cells were determined CD34^+^. Trypan blue staining was also applied to determine the cell viability, and only samples comprising > 95 % viable cells were used in further studies. Human myeloid cell line K562 was purchased from Pasteur Institute, Tehran, Iran. The cells were cultured in Roswell Park Memorial Institute (RPMI) 1640 supplemented with 10 % heat-inactivated fetal bovine serum (FBS), 2 mM L-glutamine, and 1 % antibiotics (100 U/ml penicillin and 100 mg/ml streptomycin) (Gibco BRL, Rockville, MD), and maintained at 37 °C in 5 % CO_2_ in a humidified incubator. 

### RNA isolation and qRT-PCR

Total RNA was extracted using TRIzol (Invitrogen, Carlsbad, CA) and treated with RNase-free DNase I (Fermentase, Lithuania) so as to cut down on DNA contamination. The yield and purity of the total RNA were determined using the NanoDrop 2000c spectrophotometer (Thermo Fisher Scientific, Michigan, USA). The integrity of RNA samples was also confirmed by 2 % gel electrophoresis. A total of 1 μg of RNA was used to be reverse-transcribed into cDNA using the PrimeScript 1st Strand cDNA Synthesis kit (TAKARA Bio Inc., Otsu, Japan). Quantitative real-time reverse transcription-polymerase chain reaction (qRT‐PCR) was performed using SYBR^®^ Premix Taq™ II (TAKARA, Japan) on an ABI Step One Sequence Detection System (Applied Biosystems, Foster City, CA, USA).* GAPDH* gene was used as a reference gene. For profiling the expression of miRNAs, the qRT‐PCR was performed using specific primers and a TaqMan probe according to the manufacturer's instruction (PE Applied Biosystems). To quantify the miR-155 expression level, we used the miR-Amp Kit (ParsGenome, Iran) for poly-(A)-tailing and cDNA synthesis. The expression level of the mature miRNA was determined using miR-155 specific primers as described before (Bitaraf et al., 2020[7]). The relative expression of miR-155 was normalized with U6 small nuclear RNA (U6 snRNA) as an internal control and calculated using the 2^−ΔΔCt^ method (Livak and Schmittgen, 2001[27]). 

### Transfection with miRNA mimic or inhibitor and small interfering (si)-RNA

We transfected CD34^+^ CML cells with either 50 nM of miR-155 precursor molecules (miR-155 mimics) or negative control (Ambion). In order to inhibit the miRNA function, CD34^+^ CML cells were transfected with miRCURY LNA™ microRNA inhibitor for hsa-miR-155 or its negative control (Exiqon) at a final concentration of 50 nM using lipofectamine RNAiMAX (Invitrogen) according to the manufacturer's recommendation. SMAD5 siRNA (siSMAD5) and negative control siRNA (siCtrl; Santa Cruz Biotechnology, Inc., Santa Cruz, CA, USA) were also used to silence the expression of SMAD5 in CD34^+^ CML cells. A total number of 5×10^4^ cells were transfected with siSMAD5 and siCtrl at the final concentration of 30 nmol/l using Lipofectamine RNAiMax. To assess the transfection efficiency, fluorescence microscopy was used whereby the percentage of fluorescein-labeled cells was calculated ~80 %.

### Luciferase reporter assay

Genomic DNA was extracted from the peripheral blood of healthy individuals and used as a PCR template. To construct a reporter vector, the wild-type (WT) 3ʹ-UTR sequences of SMAD5 and TGF-βR2 were cloned and inserted into psi-check2 luciferase plasmids. To study the direct interaction of hsa-miR-155 with SMAD5- or TGF-βR2- 3'-UTR, K562 cells were co-transfected with 0.4c μg of SMAD5- or TGF-βR2-3'-UTR luciferase reporter vector and 50 nM of miR-155 precursor molecule (miR-155 mimics). After forty-eight hours, the dual-luciferase assay was performed using DualGlo luciferase assay (Promega). The ratio of Renilla to Firefly luciferase was measured and normalized relative to the cells transfected by the SMAD5-3'-UTR luciferase reporter vector alone.

### Cell proliferation assays

A total number of 5×10^4^ cells/ml was transduced with either miR-155 precursor molecule or negative control, then cultured in 24-well plates and viable cells were counted from 1 to 2 days by trypan blue exclusion.

### Apoptosis assays

To assess cell death, CD34^+^ CML cells were seeded in 6-wells plates and treated with BMP, TGF-β1, miR-155 inhibitor, and their negative controls. Then, the cells were stained with Annexin V-FITC and propidium iodide (PI) (Annexin-V FITC Apoptosis Detection Kit, Sigma-Aldrich, California, USA). The stained cells were analyzed by a flow cytometer (FACScan, BD Biosciences, Heidelberg, Germany) and data were analyzed using FlowJo software (version10, Treestar, USA).

### TGF-β1 and BMP treatment of CML cells and Western blotting

CML cells were incubated with recombinant TGF-β1 (1 ng/mL) or BMP2/4 (50 ng/ mL) with or without pre-treatment with dorsomorphin (10 μM) (Sigma-Aldrich). To neutralize TGF-βR2, the cells were exposed to Anti-TGF-βR2 blocking antibody (50 μg/ mL). Then, the cells were lysed with RIPA lysis buffer and protein was isolated. A total of 100 μg of protein were separated by SDS-PAGE and transferred onto PVDF membranes. Phospho-SMAD1/5 protein levels were detected by Western blotting using SMAD1/5 antibody. GAPDH was used as loading control. The density of bands was assessed using ImageJ software. 

### Statistical analysis

All data were evaluated in triplicate against vehicle-treated control cells and collected from three independent experiments and analyzed by student's t-test. The *p-*value < 0.05 was considered to be statistically significant. All data were presented as mean ± standard deviation (SD).

## Results

### Analysis of miRNA expression in primary CML CD34^+^ versus normal BM CD34^+^ cells

**Figure 1:**
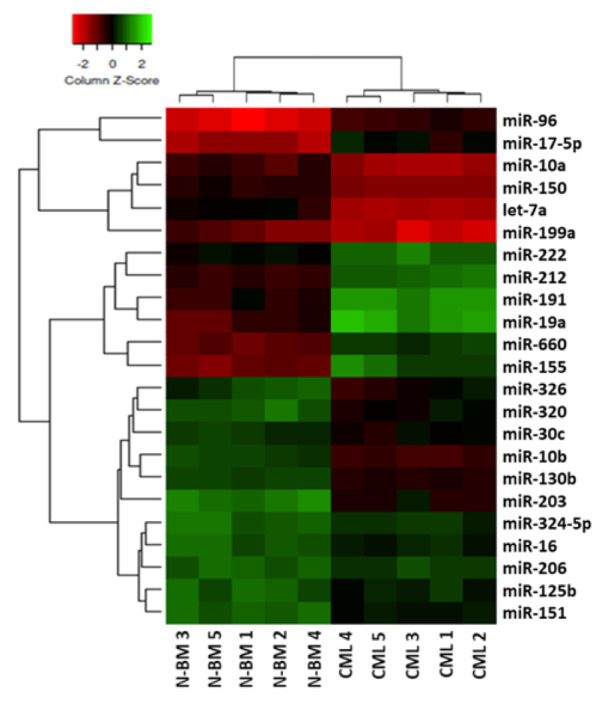
Unsupervised hierarchical cluster analysis of miRNA expression in CD34^+^ cells from CML patients and normal BM. The analysis of miRNAs differentially expressed in CD34^+^ cells from CML patients (n=5) and normal BM (N-BM) (n=5) clearly distinguished two cell groups and identified 23 differentially expressed miRNAs. The heatmap (Euclidian distance, complete linkage) represents the differential expression of miRNAs shown by green (up-regulation) versus red (down-regulation) intensity.

We profiled the expression of 47 miRNAs using miRNA TaqMan real-time PCR in primary CD34^+^ cells from CML patients (n=5) and normal individuals (n=5). Relative quantification and supervised analysis clearly distinguished two cell groups and identified 23 differentially expressed miRNAs. Among these miRNAs, 8 miRNAs were up-regulated, whereas 15 miRNAs were down-regulated in CD34^+^ CML cells compared to normal CD34^+^ BM cells (Figure 1(Fig. 1)). We also utilized bioinformatic tools for mining miRNAs, which aim at identifying the most possible miRNAs potentially involved in the survival of CML LSCs. Accordingly, among miRNAs differentially expressed in CD34^+^ cells from CML patients and normal BM, miR-155 has opted as an important miRNA that has been reported to be associated with CML, and therefore in an attempt to examine possible connection, further validation with qRT-PCR has been done.

### miR-155 is up-regulated while SMAD5 and TGF-βR2 are down-regulated in CD34^+ ^CML cells

**Figure 2:**
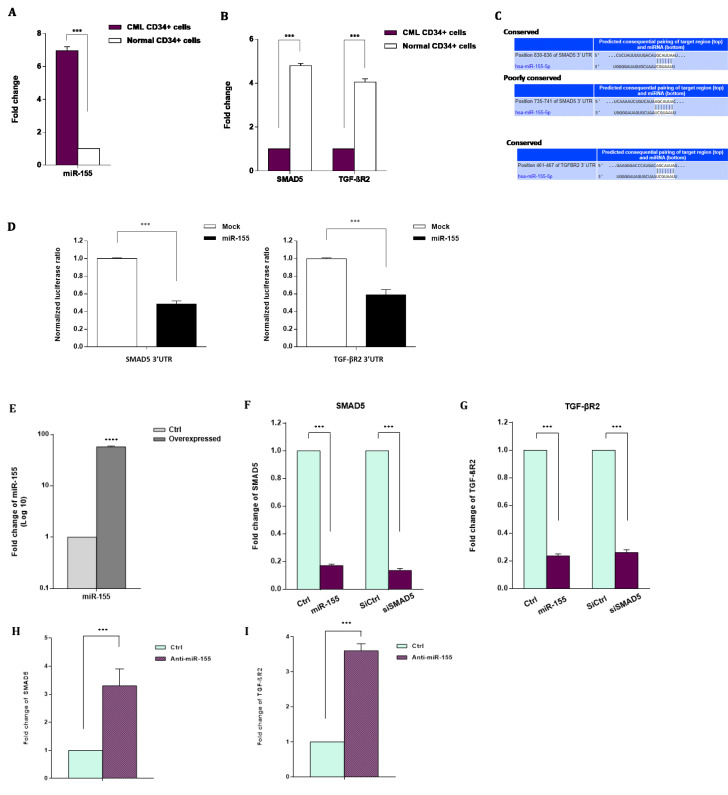
A regulatory correlation between miR-155 and TGF-β signaling pathway in CML. A. Mean expression of miR-155 in CD34^+^ cells from CML patients and normal BM. miRNA level was measured by qRT-PCR and normalized to U6 snRNA as the internal control. B. Transcript levels for SMAD5 and TGF-βR2 in CD34^+^ cells from CML patients and normal BM. Transcript levels were measured by qRT-PCR and normalized to GAPDH as a housekeeping gene. C, D. SMAD5 and TGF-βR2 are two potential direct targets of miR-155. C. Putative miR-155 binding sites in the 3'-UTR of SMAD5 and TGF-βR2. D. Relative luciferase reporter activity of K562 cells at 48 hr after co-transfection with SMAD5- or TGF-βR2-3'-UTR luciferase reporter constructs and miR-155 precursor molecule (miR-155 mimics). E. miRNA expression level evaluated by qRT-PCR in CD34^+^ CML cells after 48 hr transfection with miR-155 mimics or negative control scramble (Ctrl). F, G. SMAD5 and TGF-βR2 mRNA expression levels evaluated by qRT-PCR in CD34^+^ CML cells at 48 hr after transfection with either miR-155 mimics or negative control scramble (Ctrl). SMAD5 and TGF-βR2 mRNA expression levels in CD34^+^ CML cells 48 hr after transfection with either siRNA against SMAD5 (siSMAD5) or silencing negative control (siCtrl) is also shown. H, I. SMAD5 and TGF-βR2 mRNA expression levels evaluated by qRT-PCR in CD34^+^ CML cells at 48 hr after transfection with either anti-miR-155 or negative control scramble. Columns, mean of three different experiments; bars, SD. *** *P* < 0.001.

QRT-PCR was used to investigate the expression pattern of miR-155 in CML and normal CD34^+^ cells. In all CML cases, the mean normalized ratios for the miR-155 level were significantly higher than ratios found in healthy ones (*P* < 0.001) (Figure 2A(Fig. 2)). Moreover, because it was previously shown that a blockade in the tumor-suppressing TGF-β signals could be involved in miR-155 oncogenic function (Rai et al., 2008[41]), we sought to measure the expression levels of two key members of the TGF-β pathway (TGF-βR2 and SMAD5) in CML and normal CD34^+ ^cells. Interestingly, in all CML cases, the average normalized ratio for SMAD5 and TGF-βR2 was significantly lower than in healthy individuals (*P* < 0.001) (Figure 2B(Fig. 2)). These results indicated that CD34^+^ CML cells with high expression of miR-155 represent decreased expression levels of SMAD5 and TGF-βR2 mRNAs; therefore, we proposed that overexpression of miR-155 might be negatively correlated with low levels of SMAD5 and TGF-βR2. 

### Targeting of SMAD5 and TGF-βR2 links miR-155 to TGF-β signaling in CML

Since oncogenic miRNAs can contribute to cancer development through inducing degradation or translational repression of their target mRNAs with tumor-suppressive function, we used the TargetScan database to identify potential targets of miR-155. Among several transcripts with conserved sites in the human genome and as previous studies shown dysregulation of SMAD5 and TGF-βR2 in leukemogenesis (Tili et al., 2015[50]) we, therefore, focused on these targets. The 3'-UTR region of the SMAD5 mRNA contains two potential binding sites for miR-155 while TGF-βR2 has one putative miR-155 target site in its 3'-UTR (Figure 2 C(Fig. 2)). To confirm the miR-155 capability in SMAD5 and TGF-βR2 targeting, we used a luciferase reporter construct containing the Renilla luciferase coding gene fused with SMAD5 or TGF-βR2 3'-UTR. After co-transfection of reporter construct and miR-155 precursor molecule or negative control, it was found that miR-155 was able to specifically decrease the luciferase activity of the SMAD5- or TGF-βR2 3'-UTR reporter constructs in K562 cells (Figure 2D(Fig. 2)). These findings suggest that SMAD5 and TGF-βR2 are direct targets of miR-155. 

To investigate the impacts of miR-155 ectopic overexpression on SMAD5 and TGF-βR2 transcripts at the cellular level, the CD34^+ ^CML cells were transfected with miR-155 precursor and analyzed to assess SMAD5 and TGF-βR2 mRNA expression levels. Consistent with the reporter assays, qRT-PCR results revealed when miR-155 was overexpressed (Figure 2E(Fig. 2)), SMAD5 and TGF-βR2 mRNA expression levels were significantly lower compared to siCtrl in CD34^+^ CML cells (Figure 2F, G(Fig. 2)). Accordingly, we transfected specific siSMAD5 or siCtrl into the CD34^+^ CML cells. Our results indicated that siSMAD5 significantly reduced the expression levels of the SMAD5 and TGF-βR2 in CD34^+^ CML cells (Figure 2F, G(Fig. 2)). Given the negative correlation between miR-155 and SMAD5 and TGF-βR2, we transfected an antagomiR of miR-155 into the CD34^+^ CML cells to evaluate the miR-155 knock-down on the expression levels of SMAD5 and TGF-βR2. A significant decrease of miR-155 was observed in CD34^+^ CML cells following anti-miR-155 transfection (Supplementary Figure 1). Also, we found that the miR-155 knocking down has led to the up-regulation of SMAD5 and TGF-βR2 gene expression in CD34^+^ CML cells (Figure 2H, I(Fig. 2)). Altogether, these findings suggest that SMAD5 and TGF-βR2 might be direct potential targets of miR-155 in CD34^+^ CML cells.

### Up-regulation of miR-155 enables CD34^+^ CML cells to escape from the growth-inhibitory effects of TGF-β1 and BMP signaling

**Figure 3:**
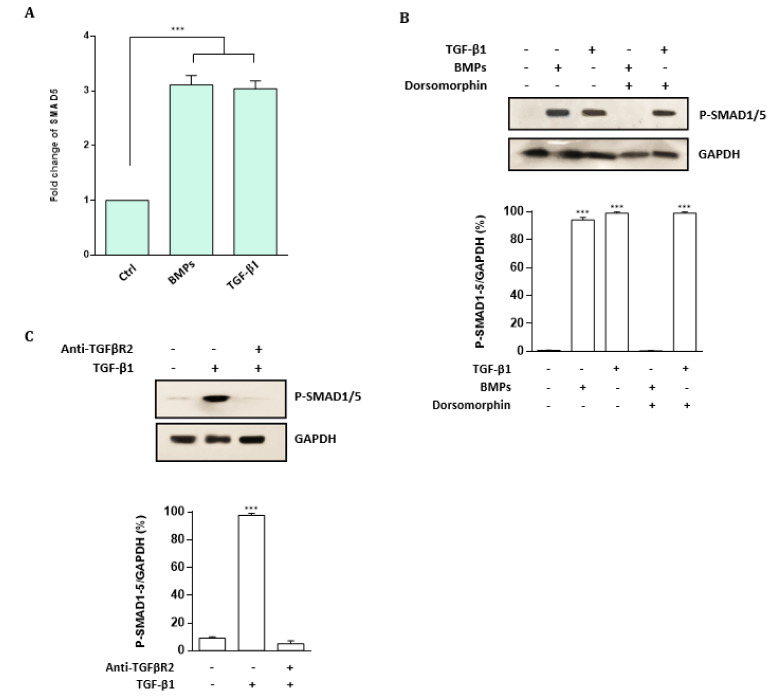
TGF-β1 and BMP signaling activate SMAD5 in CML cells. A. Fold change of SMAD5 in K562 CML cells exposed BMPs or TGF-β1 evaluated by qRT-PCR. Transcript levels were measured by qRT-PCR and normalized to GAPDH as a housekeeping gene. Columns, mean of three different experiments; bars, SD. *** *P* < 0.001. B. Western blot analysis of phospho-SMAD1/5 levels in CML cells treated with TGF-β1 or BMP2/4, with or without pre-treatment with BMP antagonist dorsomorphin. The TGF-β1 and BMP treatment induced phosphorylation of SMAD 1/5, which are significantly abolished by dorsomorphin. C. Western blot analysis of phospho-SMAD1/5 levels in CML cells treated with the blocking TGF-βR2 antibody. Results showed that TGF-β1-mediated phosphorylation of SMAD1/5 was dramatically abolished following the blocking TGF-βR2 antibody pre-treatment. All Western blot images were representative of at least three independent experiments. The density of bands was assessed using ImageJ software and represented as a relative intensity. Values are means ± SD of three independent experiments. *** *P* < 0.001.

The BMP family of cytokines can transduce the signals that are associated with different aspects of the SMAD5 functions (Massague et al., 2005[29]). The association between TGF-β1 and SMAD 1/5 has been detected in epithelial and endothelial cells (Daly et al., 2008[14]; Liu et al., 2009[26]), thus, as the first step, we sought to investigate a possible mechanism for SMAD5 activation in CML cells. Our findings revealed that not only is SMAD5 up-regulated through BMPs treatment, but TGF-β1 can also induce SMAD5 in K562 CML cells (Figure 3A(Fig. 3)). Additionally, our findings firmly cast light on BMP2/4-induced phosphorylation of SMAD1/5. Interestingly, the data substantiate the phosphorylation of SMAD1/5 after treating the K562 CML cells via TGF-β1 (Figure 3B(Fig. 3)). As BMP and TGF-β receptors are a *sine qua non *of the TGF-β1 signaling pathways (Daly et al., 2008[14]; Liu et al., 2009[26]), we used a blocking antibody anti-TGFβ-R2 and a BMP antagonist dorsomorphin. Consequentially, we unveiled that TGF-β1-mediated phosphorylation of SMAD1/5 was obliterated by pre-treatment with the blocking TGF-βR2 antibody; this suggests a significant contribution of TGF-βR2 to this process (Figure 3C(Fig. 3)). Remarkably, similar data were obtained by the time we used BMP antagonist dorsomorphin (Figure 3B(Fig. 3)). Taken together, these observations may suggest the potential of TGF-β1 and BMP signaling to activate SMAD1/5 in K562 CML cells (Figure 3(Fig. 3)).

**Figure 4:**
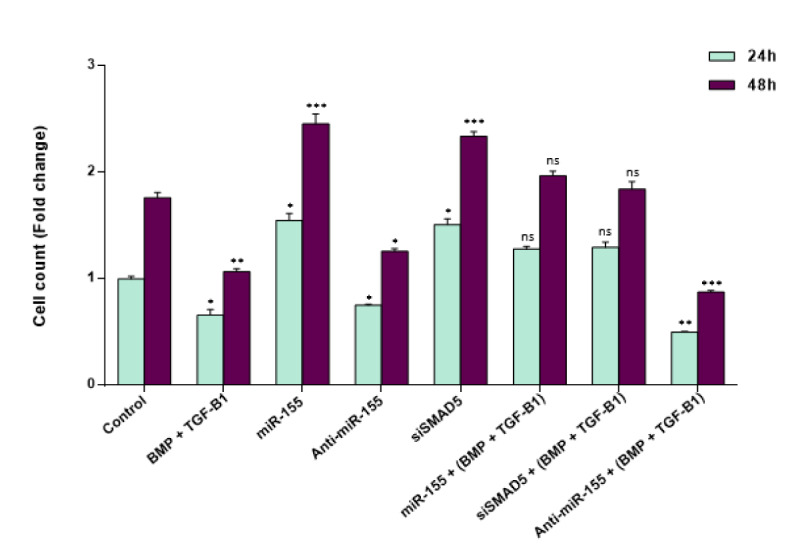
miR-155 enables CD34^+^ CML cells to escape from the anti-proliferative effect of TGF-β1 and BMP signaling. The effect of miR-155 on the cell proliferation rate of CD34^+^ CML cells at 24 and 48 hr time points. SMAD5 suppression through either miR-155 overexpression or siRNA-mediated knock-down of SMAD5 in CD34^+^ CML cells increased cell proliferation in a time-dependent manner. Additionally, anti-miR-155 induced a significant decrease in cell proliferation rate in CD34^+^ CML cells compared to controls. Importantly, down-regulation of miR-155 augmented the inhibitory effects of TGF-β1 and BMP treatment on CD34^+^ CML cell proliferation. Columns, mean of three different experiments; bars, SD. * *P* < 0.05, ** *P* < 0.01 and *** *P* < 0.001 statistically significant compared to control. ns: not significant.

To determine whether miR-155 affects cell proliferation, CD34^+^ CML cells were transfected with miR-155 precursor molecules and cell proliferation was evaluated compared to the cells transfected with negative control scramble. Our results showed that the overexpression of miR-155 by miR-155 mimics significantly promoted the proliferation rate of CD34^+^ CML cells in a time-dependent manner compared to the control group (Figure 4(Fig. 4)). This result is consistent with the oncogenic function of miR-155 in leukemic stem cells. Moreover, the down-regulation of miR-155 by anti-miR-155 resulted in reduced proliferation of CD34^+^ CML cells. Since each specific miRNA can have hundreds of direct targets, siSMAD5 is used to determine whether the increase in cell proliferation is specifically due to targeting the TGF-β pathway by miR-155 or not. Our findings demonstrated that siSMAD5 transfection had an increasing effect on CD34^+^ CML cell proliferation. This specifies that SMAD5 knock-down recapitulates the proliferative effects of miR-155 on the cells (Figure 4(Fig. 4)).

Due to the effects of TGF-β on the growth and survival of LSC (Watabe and Miyazono, 2009[53]), CD34^+^ CML cells were treated with both BMP2/4 and TGF-β1 and their inhibitory effects were evaluated within 48 hours after treatment. Interestingly, we found that pre-treatment with BMP2/4 and TGF-β1 had inhibitory effects on cell proliferation; however, the combinational treatment with miR-155 mimics resulted in a higher proliferation rate in CD34^+^ CML cells. According to our data, ectopic expression of the oncogenic miR-155 further supports CD34^+^ CML cells to evade the anti-proliferative effects of BMP and TGF-β1. Consistently, the co-treatment of siSMAD5 and BMP + TGF-β1 resulted in a higher proliferation rate in CD34^+^ CML cells compared to that of cells exposed to BMP + TGF-β1. Importantly, miR-155 down-regulation simultaneously with BMP + TGF-β1 treatment had increasing and synergistic effects on reducing the proliferation rate of CD34^+^ CML cells (Figure 4(Fig. 4)).

### Down-regulation of miR-155 augments the promoting effects of BMP and TGF-β1 on inducing apoptosis in CD34^+^ CML cells

**Figure 5:**
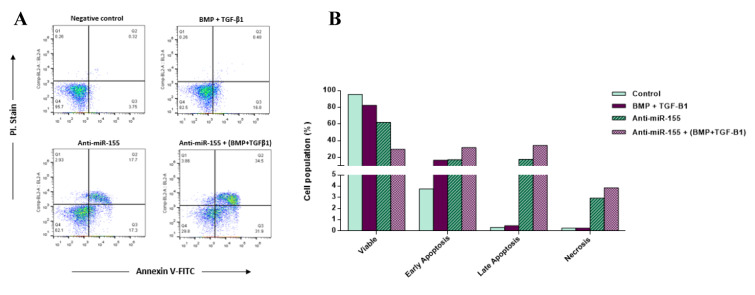
Down-regulation of miR-155 augments the promoting effects of TGF-β1 and BMP signaling on inducing apoptosis in CD34^+^ CML stem cells. TGF-β1 and BMP treatment and transfection of anti-miR-155 promoted apoptosis in CD34^+^ CML cells compared to controls. Importantly, data revealed that TGF-β1 and BMP treatment and miR-155 down-regulation may act in a synergistic manner to exert their pro-apoptotic effects on CD34^+^ CML cells. A. Annexin-PI histograms illustrating apoptotic cells (Annexin V-positive/PI-negative and Annexin V-positive/PI-positive) versus necrotic cells (Annexin V-negative/PI-positive) in CD34^+^ CML cells following transfection with negative control, anti-miR-155, and treating with TGF-β1 and BMP. The assay was carried out 48 hr after incubation. The X-axis displays Annexin-V-fluorescein isothiocyanate (FITC) and the Y-axis displays propidium iodide (PI). B. The percentage of viable cells, early apoptosis, late apoptosis and necrosis of CD34^+^ CML cells following transfection with anti-miR-155 and TGF-β1 and BMP treatment at 48 hr.

As there are some reports regarding the apoptotic effects of TGF-β signaling on LSCs, the CD34^+^ CML cells were pre-treated with BMP and TGF-β1 and stained with Annexin-V-FITC and Propidium Iodide (PI). Flow cytometry analysis illustrated an apoptotic effect of BMP and TGF-β1 treatment within 48 hours after treatment (Figure 5(Fig. 5)). The results also showed that the down-regulation of miR-155 mediated by anti-miR-155 induced further apoptosis than the control group in CD34^+^ CML cells which is consistent with the oncogenic function of miR-155 in leukemia stem cells. As shown in Figure 5,(Fig. 5) anti-miR-155 transfection induced apoptosis up to 35 %, dividing into 17.3 % and 17.7 % early and late apoptosis, respectively. More importantly, transfection of anti-miR-155 into CD34^+^ CML cells pre-treated with BMP and TGF-β1 induced 66.4 % of cell apoptosis in 48 hours. This result further supports the synergistic effects of anti-miR-155 and BMP combinatorial therapy to induce apoptosis in CD34^+^ CML cells (Figure 5(Fig. 5)).

## Discussion

As an important group of non-coding RNAs, miRNAs play a critical role in the regulation of gene expression at the post-transcriptional level (Thomson et al., 2006[49]). Numerous studies have investigated the expression and function of miRNAs in the CML progression, but further studies need to cast light on the role of the specific miRNAs involved in regulating the self-renewal properties of LSCs (Babashah et al., 2012[5]). miRNAs can *per se* determine the cell fate by interacting with other molecules in signaling pathways (Peng and Croce, 2016[39]). In other words, they can put the cells on long-run viability or even promote their apoptosis. 

Since in many cancers, the aberrant miRNA expression is an undeniable characteristic, using miRNA expression profiling is beneficial for better evaluation of diagnostic and therapeutic values (Babashah et al., 2012[5]). Herein, we reported an miRNA profile expression in the CD34^+ ^CML cells led to the identification of 8 up-regulated miRNAs -including miR-19a, miR-17-5p, miR-191, miR-96, miR-155, miR-660, miR-212, and miR-222-but 15 down-regulated miRNAs (i.e. miR-10a, miR-150, miR-151, let-7a, miR-199a, miR-326, miR-320, miR-30c, miR-10b, miR-130b, miR-203, miR-324-5p, miR-16, miR-206, and miR-125b) **(Figure 1**(Fig. 1)**)**.

Mi et al. suggested that different miRNAs in the miR-17-92 cluster may participate critically in the development of *MLL*-rearranged acute leukemia, by negatively regulating targets that are *per se* positive modulators of cell differentiation and apoptosis, or negative regulators of cell proliferation (Mi et al., 2010[31]). Some scrapes of evidence bear out that miR-19a - an important oncogenic member of miR-17-92 cluster - was aberrantly up-regulated in different kinds of human cancers, particularly leukemia; this miRNA has been also detected to be bona fide associated with the prognosis of cancer patients (Peng et al., 2019[40]). By inhibiting apoptosis, miR-19 is sufficient for promoting c-Myc-induced lymphomagenesis. The oncogenic characteristic of miR-19 is imputed to its well-documented roles in repressing the tumor suppressor PTEN. Consistently, miR-19 functions to activate the Akt-mTOR - as a mammalian target of the rapamycin - pathway, so functionally antagonizing PTEN to promote cell survival (Olive et al., 2009[36]).

The core-binding factor (CBF) as a key hematopoietic transcription factor controls the transcription of different genes that *ipso facto* involve in both embryonic and post-embryonic hematopoietic development. Interestingly, Fischer et al. demonstrated that miR-17 as a regulator of RUNX1 (encoding a CBF subunit) can up-regulate the KIT expression. Consequently, they proposed that miR-17 can mimic the effects of CBF-AML fusion proteins by influencing a core RUNX1-miRNA mechanism of KIT-induced proliferation of undifferentiated myeloid cells (Fischer et al., 2015[16]).

Previously, it has been identified that out of 157 candidate miRNAs that were tested, miR-10a, miR-150, and miR-151 were down-regulated in CML cells, whereas miR-96a was up-regulated in those putative cells. Importantly, this study showed that the expression of miR-150 and miR-151 was decreased due to the BCR-ABL expression; however, down-regulation of miR-10a was independent on BCR-ABL1 activity and in turn caused increasing cell growth of CML cells by stimulating upstream stimulatory factor 2 (USF2) (Agirre et al., 2008[1]).

Jose-Eneriz et al. using the expression profiling of imatinib mesylate-resistant and -responder CML patients cast light on a group of 19 miRNAs differentially expressed, among which, miR-191 was up-regulated in resistant CML patients (San Jose-Eneriz et al., 2009[46]). To grasp the importance of miRNAs that may share a common role in leukemogenesis, Xiong et al. performed an exhaustive miRNome analysis of human myeloid leukemia cells that *ipso facto* showed the up-regulation of miR-191 and miR-25 in myeloid leukemia cells (Xiong et al., 2014[55]).

The up-regulation of miR-29a-3p and miR-660-5p has been remarkably identified in a miRNA expression profiling of different subpopulations of CML stem cells. Interestingly, this up-regulation can lead to the down-regulation of their respective targets - TET2 and EPAS1 - and confer TKI-resistance to CML stem cells. Accordingly, these findings can show that the aberrant miRNA expression in CML stem cells could justify the observed intrinsic TKI-resistance in such cell populations (Salati et al., 2017[45]).

miR-155, a product of the B-cell integration cluster gene (BIC), is a key regulator of inflammation and expressed in hematopoietic stem cells and mature hematopoietic cells including activated B-cells, T-cells, monocytes, and macrophages (Alivernini et al., 2018[2]). Overexpression of miR-155 has been reported in several human cancers including breast, cervical, hepatocellular, colorectal, B-cell lymphoma, and chronic lymphocytic leukemia (CLL) (Fu et al., 2017[17]; Gao et al., 2018[18]; Mattiske et al., 2012[30]; Papageorgiou et al., 2017[37]; Wu et al., 2018[54]; Zhang et al., 2018[58]). Chen et al. revealed that the phosphorylation and activation of signal transducers and activator of transcription 6 (STAT6) up-regulates miR-155 expression to develop the pathogenesis of CLL (Chen et al., 2019[11]). Beyond that, overexpression of miR-155 can enhance B-cell receptor signaling and also is associated with aggressive disease in CLL (Cui et al., 2014[13]). Using *in vivo* models of acute myeloid leukemia, it has been shown that miR-155 can also promote FLT3-ITD-induced myeloproliferative in the bone marrow, spleen, and blood by suppressing the interferon response (Wallace et al., 2017[52]). In breast cancer cells, high expression of miR-155 could accelerate cancer progression through down-regulating cell adhesion molecule 1 (CADM1) (Zhang et al., 2019[56]). miR-155 can also promote hepatocellular carcinoma cell proliferation *in vitro* and tumorigenesis *in vivo* by inhibiting F-box and WD repeat domain containing 7 (FBXW7) expression (Tang et al., 2016[48]). 

SMADs are a group of proteins belonging to the TGF-β superfamily of modulators. TGF-β signaling is transduced by SMAD proteins regulates several cellular functions such as proliferation, differentiation, and apoptosis (Liu et al., 2003[25]). Previous *in vivo* or *in vitro* findings have demonstrated that TGF-β can suppress the proliferation of the most primitive types of hematopoietic cells. For example, SMAD5 through TGF-β1 and TGF-β2 inhibits the proliferation of hematopoietic progenitor cells in human adult bone marrow (Bruno et al., 1998[8]). Accordingly, SMAD5 has been suggested as a link for miR-155-mediated regulation of the TGF-β pathway and lymphomagenesis in diffuse large B cell lymphoma (Rai et al., 2010[42]). 

In the present study, we identified miR-155 in a group of highly expressed miRNAs in CD34^+^ CML cells. Since it was previously demonstrated that a blockade in the tumor-suppressing TGF-β signals could contribute to miR-155 oncogenic function (Rai et al., 2008[41]), we investigated whether up-regulation of miR-155 enables CD34^+^ CML cells to escape from the growth-inhibitory effects of TGF-β1 signaling or not. The results revealed a significantly high expression of miR-155 in CD34^+^ CML cells compared to the normal BM CD34^+^ cells (Figure 2A(Fig. 2)). Our findings confirmed the previous reports regarding the oncogenic functions of miR-155 in cancer cells. Since it has been suggested that TGF-β signaling is essential for regulating the survival of CML stem cells (Naka and Hirao, 2017[32]), we sought to measure the expression levels of two key members of the TGF-β pathway (SMAD5 and TGF-βR2) in CD34^+ ^CML and normal cells. We found that down-regulation of SMAD5 and TGF-βR2 was associated with increased expression of miR-155 in CD34^+ ^cells from CML patients compared to healthy controls (Figure 2A, B)(Fig. 2). Consistently, we found that SMAD5 is negatively regulated by the oncogenic miR-155 (Figure 2C, D(Fig. 2)). These findings led us to propose that dysregulation of TGF-β signaling may be caused by miR-155 up-regulation.

BMPs are another member of the TGF-β superfamily which exert their multiple biological effects through transmembrane BMP type I (BMPRI) and type II receptors (BMPRII). Three members of the SMAD family including SMAD1, SMAD5, and SMAD8 can become phosphorylated and activated by BMPRI (Nishimura et al., 2003[35]). Multiple studies have indicated the apoptotic and anti-proliferative roles of BMPs in various types of cancer cells. BMP-dependent promotion of apoptotic cell death in human pulmonary artery smooth muscle cells has been reported by activation of caspases-3, -8, and -9 (Lagna et al., 2006[24]). In human myeloma cell lines, BMP2 induces apoptosis by the accumulation of p21 CIP1/WAF1 and p27 KIP1 in the G1 phase of the cell cycle (Kawamura et al., 2002[23]). In a recent study, BMP2 has been shown to have pro-apoptotic effects on glioma-initiating cells by modulating the erythropoietin-producing hepatocellular carcinoma receptor A6 (EPHA6) (Raja et al., 2019[43]). In the early stages of breast cancer, BMP2 significantly inhibited the proliferation rate of the cancer cells and promoted apoptosis through G1 arrest in the cell cycle (Chen et al., 2012[10]). BMP2 and BMP4 were down-regulated in hematopoietic cells from CML patients compared to healthy individuals (Gerber et al., 2013[19]). In line with these results, Toofan et al. showed that BMP2, BMP4, and activin A were all down-regulated in chronic phase CML (Toofan et al., 2014[51]). 

In the present study, we demonstrated that TGF-β1 and BMP signaling activate SMAD1/5 in CML cells (Figure 3(Fig. 3)). We also identified that TGF-β1 and BMP treatment has anti-proliferative and pro-apoptotic effects on CD34^+ ^CML cells. Moreover, we showed that miR-155 plays an important role in CML pathogenicity by enforcing CD34^+^ cells to escape from the growth-inhibitory effects of TGF-β1 and BMP signaling. Also, to further demonstrate that the increase in cell proliferation rate observed upon miR-155 overexpression in CD34^+^ CML cells resulted specifically from SMAD5 down-regulation, we transfected CD34^+^ CML cells with siRNA against SMAD5. In doing so, we found an increase in cell proliferation rate. These observations strongly propose that the proliferative effects of miR-155 can be attributed to the targeting of SMAD5 (Figure 4(Fig. 4)). These findings may provide a piece of evidence for a direct link between TGF-β/Smad5 and miR-155 in CML pathogenesis. Furthermore, we found that down-regulation of miR-155 induced a significant decrease in cell proliferation rate and promoted apoptosis in CD34^+^ CML cells compared to controls. Most importantly, our data revealed that TGF-β1 and BMP treatment and miR-155 down-regulation may act synergistically to exert further anti-proliferative and pro-apoptotic effects on CD34^+^ CML cells (Figures 4(Fig. 4), 5(Fig. 5)).

In conclusion, our study showed that the expression of miR-155 is up-regulated in CD34^+^ CML cells and revealed a regulatory correlation between miR-155 and TGF-β/SMAD pathway. Our findings primarily demonstrated that up-regulation of miR-155 rescues CD34^+^ CML stem cells from the anti-proliferative effect of TGF-β1 and BMP signaling. In contrast, miR-155 down-regulation augments the promoting effects of TGF-β1 and BMP on inducing apoptosis in CD34^+^ CML stem cells. Obviously, further studies will be necessary to identify other miR-155 targets and the exact molecular mechanisms to manipulate this miRNA as a promising therapeutic option for CML.

## Acknowledgements

We would like to thank Dr. Majid Mossahebi-Mohammadi from Wenzhou Medical University, China and Dr. Amirreza Bitaraf and Dr. Ehsan Razmara from Tarbiat Modares University, Iran for their excellent technical assistance and advice. 

## Conflict of interest

The authors declare no conflict of interest.

## Supplementary Material

Supplementary information
