# Supplementary material for: Up-regulation of miR-155 potentiates CD34+ CML stem/progenitor cells to escape from the growth-inhibitory effects of TGF-ß1 and BMP signaling
Source: EXCLI J. 2021 Apr 15;20:748–63. doi: 10.17179/excli2021-3404 (PMC8073837; doi:10.17179/excli2021-3404)
Supplement: Supplementary information [file EXCLI-20-748-s-001.pdf]

EXCLI Journal 2021;20:748-763 – ISSN 1611-2156
Received: January 17, 2021, accepted: April 08, 2021, published: April 15, 2021

Supplementary information to:

Original article:

UP-REGULATION OF MIR-155 POTENTIATES CD34$^+$ CML STEM/PROGENITOR CELLS TO ESCAPE FROM THE GROWTH-INHIBITORY EFFECTS OF TGF-B1 AND BMP SIGNALING

Touba Mahdloo[1], Pantea Sahami[2], Reihaneh Ramezani[2], Mojtaba Jafarinia[1], Hamedreza Goudarzi[1], Sadegh Babashah[3,*]

[1] Department of Genetics, Faculty of Basic Sciences, Islamic Azad University, Marvdasht, Iran
[2] Department of Biomedical Sciences, Women Research Center, University of Alzahra, Tehran, Iran
[3] Department of Molecular Genetics, Faculty of Biological Sciences, Tarbiat Modares University, Tehran, Iran

* **Corresponding author:** Sadegh Babashah, Ph.D., Department of Molecular Genetics, Faculty of Biological Sciences, Tarbiat Modares University, P.O. Box: 14115-154, Tehran, Iran, Tel.: +98 21 8288 4468, fax: +98 21 8288 4717,
E-mail: sadegh.babashah@gmail.com; babashah@modares.ac.ir

http://dx.doi.org/10.17179/excli2021-3404

This is an Open Access article distributed under the terms of the Creative Commons Attribution License (http://creativecommons.org/licenses/by/4.0/).

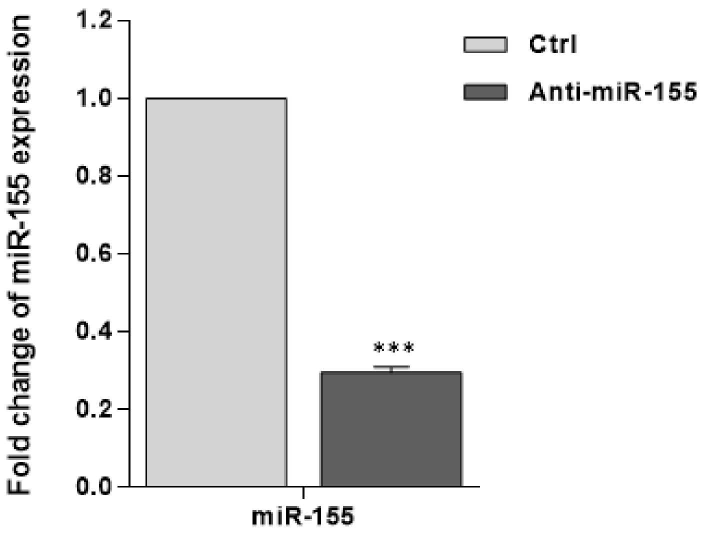

Supplementary Figure 1: Fold change of miR-155 in CD34$^+$ CML cells following transfection with anti-miR-155. miRNA level was measured by qRT-PCR and normalized to U6 snRNA as internal control.
